# E-liquid alters oral epithelial cell function to promote epithelial to mesenchymal transition and invasiveness in preclinical oral squamous cell carcinoma

**DOI:** 10.1038/s41598-023-30016-0

**Published:** 2023-02-27

**Authors:** Jefferson Muniz de Lima, Carolina Carneiro Soares Macedo, Gabriela Vasconcelos Barbosa, Lúcio Roberto Cançado Castellano, Michael P. Hier, Moulay A. Alaoui-Jamali, Sabrina Daniela da Silva

**Affiliations:** 1 Department of Otolaryngology Head and Neck Surgery, Lady Davis Institute for Medical Research, Sir Mortimer B. Davis-Jewish General Hospital, McGill University, Montreal, QC Canada; 2 Segal Cancer Centre and Lady Davis Institute for Medical Research, Sir Mortimer B. Davis-Jewish General Hospital, Departments of Medicine, Oncology, and Pharmacology and Therapeutics, Faculty of Medicine, McGill University, Montreal, QC Canada; 3 Department of Oral Pathology, Faculty of Dentistry, Federal University of Paraiba (UFPB), João Pessoa, Paraíba Brazil; 4 Human Immunology Research and Education Group, Federal University of Paraíba, João Pessoa, Paraíba Brazil; 5 Graduate Program in Dentistry, Federal University of Paraíba, João Pessoa, Paraíba Brazil

**Keywords:** Head and neck cancer, Metastasis, Oral cancer

## Abstract

The gaining popularity of tobacco and nicotine delivery products, such as electronic cigarettes (e-cigarettes) being perceived as relatively safe is of a medical concern. The long-term safety of these new products remains uncertain for oral health. In this study, in vitro effects of e-liquid were assessed in a panel of normal oral epithelium cell lines (NOE and HMK), oral squamous cell carcinoma (OSCC) human cell lines (CAL27 and HSC3), and a mouse oral cancer cell line (AT84) using cell proliferation, survival/cell death, and cell invasion assays. In addition, signaling pathways underlying the pro-invasive activity of e-cigarettes were evaluated by gene and protein expression analysis. We demonstrated that e-liquid promotes proliferation and anchorage-independent growth of OSCC and induces morphological changes associated with enhanced motility and invasive phenotypes. Furthermore, e-liquid-exposed cells express significantly reduced cell viability, regardless of e-cigarette flavour content. At the gene expression level, e-liquid induces changes in gene expression consistent with epithelial to mesenchymal transition (EMT) revealed by reduced expression of cell epithelial markers such as E-cadherin and enhanced expression of mesenchymal proteins like vimentin and B-catenin seen both in OSCC cell lines and normal oral epithelium cells. In summary, the ability of e-liquid to induce proliferative and invasive properties along the activation of the EMT process can contribute to the development of tumorigenesis in normal epithelial cells and promote aggressive phenotype in pre-existing oral malignant cells.

## Introduction

Oral squamous cell carcinoma cancer (OSCC) is a common cancer worldwide with approximately 600,000 new cases diagnosed per year^1,2^. This neoplasia is characterized by local aggressiveness and a high incidence of tumor relapse and metastasis (18–76% in 14 months) with poor survival rates (42% in 3 years)^3^. OSCC is a complex and heterogeneous disease with multifactorial etiologies associated with tumor development^4–6^. Historically, the traditional risk factors for OSCC were attributed to excessive tobacco smoke and alcohol consumption^4–6^. For decades, public health agencies have been promoting anti-smoking campaigns to provide public awareness and the need for an effective population-wide strategy for preventing smoking uptake^7–10^. At present, it is a concern that different alternatives for tobacco and nicotine delivery, such as electronic-cigarettes (e-cigarettes) products have been gaining massive popularity worldwide especially among young adults^11^.

E-cigarettes are battery-operated devices containing cartridges filled with nicotine and primary products of the oxidative stress process, including nitrosamines 4-(methylnitrosamine), -1-(3-pyridyl), -1-butanone (NNK), N'-nitrosonornicotine (NNN), aldehydes, phenolic compounds, polycyclic aromatic hydrocarbons, tobacco alkaloids, heavy metals and flavours^12–16^. Evidence showed these compounds are potent carcinogens able to cross the placenta and cause fetal tissue genotoxic damage^17–19^. When an e-cigarette is used, the e-liquid is turned into a vapor or steam that is inhaled by the smoker. Since the recent introduction of e-cigarettes, only limited research has tackled their long-term health risk impact^12,20–24^. Although the levels of many identified chemicals and metals present in e-cigarettes are considered, at lower levels, comparable to tobacco smoke produced by conventional cigarettes, these compounds generate DNA-reactive carcinogens (genotoxins) that may act as an important mediator of cell transformation under oxidative stress^17–22,25–27^. In particular, e-cigarettes aerosols are produced in the vicinity of the oral mucosal epithelium making this tissue a major target under risk of developing oral cancer in e-cigarettes’ users. In this study, we demonstrated that e-cigarettes induce morphological and behaviour changes in normal oral keratinocytes, as well as they can promote progression of existing tumors by inducing epithelial to mesenchymal transition (EMT) signaling, which enhances the invasive abilities of OSCC.

## Materials and methods

### E-liquid characterization

**Figure 1:**
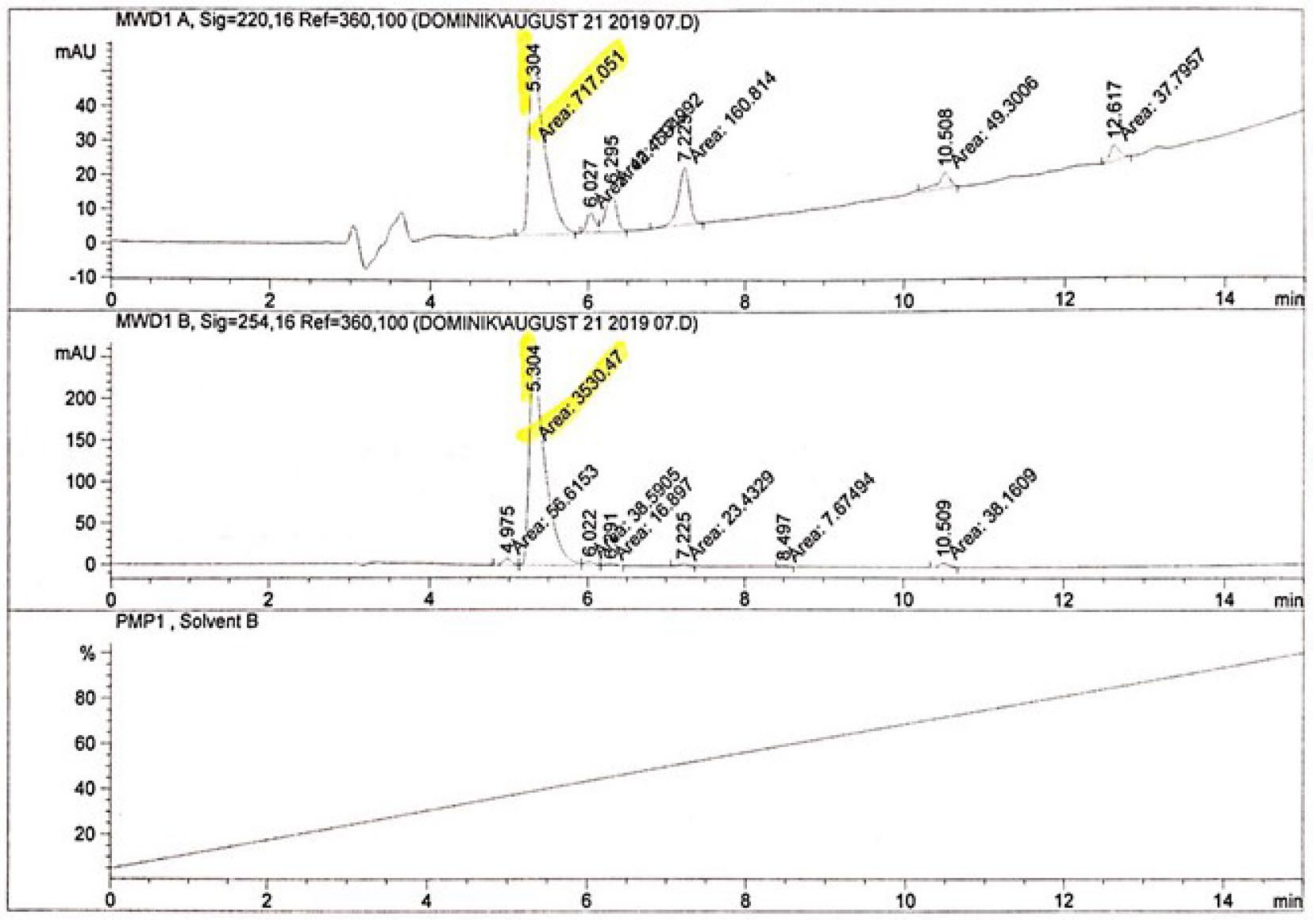
High-pressure liquid chromatography (HPLC) confirms the nicotine content of e-liquid before cell treatment. The graphs represent the composition of chemicals in the e-liquid solution, pure nicotine and methanol (used as control). The mean (± SD) of three determinations relative to nicotine content within the e-liquid was reported to be 0.73 ± 1 mg/mL. (SD = standard deviation). HPLC: high-pressure liquid chromatography.

The nicotine level in the e-liquid was measured by high-pressure liquid chromatography (HPLC) normalized with 1 mg/mL nicotine (Aldrich Chemical, N3876) in methanol solution. The commercial e-liquid (Le Patriote -flavoured and unflavoured e-juice) consisted of 20 mg/mL nicotine in glycerine and propylene glycol solution (70–30%). The progress of the reactions and the homogeneity of the products were monitored by analytical HPLC Agilent 1200 series instrument, using BDS-Hypersil-C18 reverse phase column, 4.6 × 250 mm, particle size 5 mm, pore 120 Å; solvent A: 0.1% TFA/water, solvent B: 0.1% TFA/acetonitrile; gradient from 0% B to 100% B in 15 min; flow 1 mL/min; injection volume 5μL. Samples were read in HPLC in triplicates before use in cell culture to confirm the content of the e-liquid and maintain the accuracy of the concentration in preclinical assays (Fig. 1).

### Cell culture

The spontaneously immortalized but non-malignant human oral mucosal keratinocytes (HMK) was kindly provided by Dr. Tuula Salo (University of Helsinki). The HMK were cultured in Keratinocytes-SFM medium (GIBCO, 17005075) supplement with L-glutamine and bovine pituitary extract. In addition, normal epithelial cells (NOE) isolated from human tongue was maintained in cell culture with KSF serum-free medium supplemented with 5 µg/mL of bovine pituitary extract (Gibco/Invitrogen Life Technology, Carlsbad, CA, USA) as we previously described^28^. The oral human cancer cell lines CAL27 (ATCC® CRL-2095™, American Type Culture Collection) and HSC3 (JCRB 0623, a human tongue squamous cell carcinoma cell line, Osaka National Institute of Health Sciences, Japan) were maintained in DMEM (Corning, 10-017-CV) supplemented with 10% FBS (Wisent Bioproducts, 080–150) with 1% penicillin and streptomycin antibiotics (Corning Inc., 30-002-CI). The mouse oral cancer cell line (AT84) was established and characterized by our group^29^ and cultured in RPMI 1640 (Fisher Scientific, 11-875-093) supplemented with 10% FBS and 1% penicillin and streptomycin antibiotics (Corning Inc., 30-002-CI). Cells were cultured at 37 °C with 5% CO_2_. Biological replicates were done for all in vitro assays in six independently repeated experiments performed on cells of the same cell line but derived from a biologically distinct source or of a different passage. The cell lines were tested for mycoplasma contamination (MycoZAP; Lonza, NJ, USA).

### In vitro cell cytotoxicity

The acute cytotoxicity effect of e-liquid was assessed using the 3-(4,5-dimethylthiazol-2-yl)-2,5-diphenylte-trazolium bromide (MTT) assay^30^. Cells were seeded at a density of 5 × 10^4^ cells/mL in a 96-well plate and treated with e-liquid content ranging from 10 to 0.0001% (v/v) (2 mg/mL, 200 ug/mL, 20 ug/mL, 2 ug/mL, 0.02 ug/mL) for 24 h, 48 h and 7 h at 37 °C. Cell medium was changed to serum free medium (SFM) and 100μL of MTT solution (Sigma Aldrich Corporation) was added. Plates were incubated at 37 °C for 4 h in the dark, and the purple formazan crystals were dissolved in 100 μL dimethyl sulfoxide (DMSO) for 5 min on orbital shaking. The color intensity was quantified by optical density on FLUOstar OPTIMA microplate reader (BMG Labtech, Ortenberg, Germany) at 560 nm. Data are presented as mean absorbance *versus* medium control and represent the percentages of viable cells compared to the survival of a control group.

### Clonogenic assay

The chronical cytotoxicity effects of e-liquid on tumor cell proliferation and survival were assessed using the colony formation assay^30^. The cell lines CAL27, HSC3 (600 cells/well) and AT84 (500 cells/well) were plated in triplicates in a 6-well plate. After 6 h of incubation, cells were treated with 10% and 1% (v/v) e-liquid solutions for additional 48 h of incubation, then cells were washed with phosphate buffer solution (PBS) and stained with 0.05% (w/v) crystal violet (Sigma Aldrich Corporation, C3886) and 4% formaldehyde solution (v/v) (Sigma Aldrich Corporation, 8.18708). The colonies > 100 μm in diameter were counted using a colony counter (Oxford Optronix Gelcount, Inc., Milton Park, Abingdon, UK).

### Spheroid preparation and evaluation

Spheroid cell culture approach was used to evaluate phenotypic changes and biological signaling pathways that might be disrupted following exposure to the e-liquid. The spheroid medium was prepared using DMEM-F12 (3:1, Invitrogen) containing 2% B27 supplement (Invitrogen), and 20 ng/mL epidermal growth factor (Sigma-Aldrich). Tissue culture dishes were coated with a polyhydroxyethylmethacrylate polymer (polyHEMA, Sigma-Aldrich) and spheroids were maintained with DMEM-F12 (3:1, Invitrogen) supplemented with 2% B27 (Invitrogen) and 20 ng/mL epidermal growth factor (Sigma-Aldrich). Briefly, polyHEMA was dissolved in 95% ethanol at 12% (w/v) and the working solution was prepared by a further dilution of 1:10 in 95% ethanol and added to 24-well plates at 0.1 mL per well. A hydrophobic surface was formed after the polyHEMA solution dried out at room temperature in a tissue culture hood. HSC3 and CAL-27 single cell suspensions were seeded at two dilutions of 3 × 10^2^ and 1 × 10^3^ cells/mL per well in 6 replicates. 96 h after spheroid formation, supernatant was removed and replaced by fresh culture media alone or with 1% and 0.1% e-liquid solutions and spheroids were maintained for 96 h in a humidified incubator at 37 °C and 5% CO_2_ atmosphere. The spheroids were photographed with an inverted microscope (EVOS FL cell imaging system, Life Technologies), the number of spheres (> 50 μm) was counted and the diameters of each spheroid was measured and quantified by using a macro for automated spheroid size analysis from ImageJ Software (version 1.50i, National Institutes of Health (NIH), Bethesda, MD). Representative images were generated from the same seeding-concentration.

### Cell migration assay

Wound healing migration assay was based on methodology previously described^30^. The areas were measured in triplicates and photographed with an inverted microscope (EVOS FL cell imaging system, Life Technologies) at 0 and 24 h post-incubation. Quantification of the wound healing percent closure was determined using the tool called MRI-Wound Healing Tool macro ((http://dev.mri.cnrs.fr/projects/imagej-macros/wiki/Wound_Healing_Tool) from ImageJ (version 1.50i, National Institutes of Health (NIH), Bethesda, MD). Statistical significance was analyzed using the Student’s t test.

### Invasion assay

Cell invasion was quantified using 8 μm porous chambers coated with BD Matrigel Matrix (BD Biosciences, Bedford, MA, USA) according to the manufacturer’s recommendations. Cells were fixed with 4% (v/v) formaldehyde, stained with 0.05% (w/v) crystal violet, and photographed with an inverted microscope (EVOS FL cell imaging system, Life Technologies). Cells were counted in ten random fields using the cell counting tool from ImageJ (version 1.50i, National Institutes of Health (NIH), Bethesda, MD). Each experiment was performed at least three times and results are expressed as average ± SD. Statistical significance was analyzed using Student’s t test^30^.

### Immunoblotting

Sub-confluent cells were washed with PBS, lysed in RIPA lysis and extraction buffer (50 mM Tris–HCl, pH7.5, 150 mM NaCl, 1% NP40, 0.1% SDS, 5 mM EDTA, 25 mM sodium deoxycholate) supplemented with 1 mM PMSF, 1 mM NaF and protease inhibitor cocktail (Roche, 4693159001) for 15 min on ice and centrifuged (14,000xG at 4 °C for 20 min) to separate the cell lysate. Cell concentration was measured by the Bradford protein assay and then added with SDS sample buffer (17 mM Tris, pH 6.8, 30% glycerol, 10% SDS, 0.02% bromophenol blue, 6% β-mercaptoethanol) and denatured for 5 min. Samples (20 μg) were then resolved through 15% SDS-PAGE gels, transferred to polyvinylidene fluoride (PVDF) membrane (MerckMillipore, IPVH00010), blocked with TBST with 5% w/v non-fat dry milk, blotted overnight with specific antibodies (Table 1) overnight at 4 °C, and then amplified with horseradish peroxidase-conjugated secondary antibodies for 1 h at room temperature and enhanced by chemiluminescence detection systems (Thermo Fisher Scientific, 32106 and 34095). Band densitometry was performed using ImageJ (version 1.50i, National Institutes of Health (NIH), Bethesda, MD).

**Table 1:** Antibodies used in this study to evaluate protein expression by western blot.

Name	Source	Company	Dilution
E-cadherin	Rabbit pAB	Abcam	1:1000
B-catenin	Rabbit pAB	Cell Signalling	1:1000
Vimentin	Mouse mAB	Cell Signalling	1:500
GAPDH	Rabbit	Sigma Aldrich Corp	1:10,000
Anti-mouse IgG-peroxidase-conjugated	Goat	Bio-Rad Laboratories	1:5000

*GAPDH* glyceraldehyde 3-phosphate dehydrogenase, *IgG* immunoglobulin G.

### Quantitative RT-PCR

Fifty thousand cells were plated in 6-well Corning® Costar® plates. Cells were either exposed or not exposed to e-liquid. RNA was extracted 24 h later using total RNA Mini Kit (Qiagen®) according to the manufacturers' instructions. cDNA was synthesized from 500 ng of RNA using Superscript III reverse transcriptase (Invitrogen®) and oligo-dT primers (Invitrogen®). qRT-PCR was performed in the ABI PrismTM 7900 (Applied) using SYBR® Green (Applied) in a 10μL total volume and quality controls were used according to the MIQE Guidelines. The reactions were carried out in triplicate. GAPDH, ACTB and HPRT were used as endogenous controls (Table 2).

**Table 2:** Primers used in this study to evaluate gene expression by quantitative RT-PCR.

Name	Forward	Reverse	T °C
*CDH1*	CTTGAGCCAGCTGCACAG	GTGGGGTCAGTATCAGCC	62
*VIM*	CTCCCTCTGGTTGATAC	CAAGGTCATCGTGATGC	62
*CTNNB1*	CTTCAGAACAGAGCCAATG	GAGTGAAAAGAACGATAGCTAG	63
*HPRT*	GAACGTCTTGCTCGAGATGTGA	TCCAGCAGGTCAGCAAAGAAT	60
*GAPDH*	GAAGGTGAAGGTCGGA	GGGTCATTGATGGCAAC	63
*ACTB*	GCACCCAGCACAATGAAG	CTTGCTGATCCACATCTGC	64

### Immunocytochemistry

Immunocytochemistry was carried out as described earlier^20^. Incubations with the primary antibodies diluted in PBS were conducted overnight at 4 °C for: anti-e-cadherin (Cell Signalling, 1:250) and anti-vimentin (Cell signalling, 1:200). The sections were washed and incubated with secondary antibodies (Advanced TM HRP Link, DakoCytomation, K0690, Denmark) for 30 min followed by the polymer detection system (Advanced TM HRP Link, DakoCytomation) for 30 min at room temperature. Reactions were developed with a solution containing 0.6 mg/mL of 3,3′-diaminobenzidine tetrahydrochloride (DAB, Sigma, St Louis) and 0.01% H_2_O_2_ and then counter-stained with Mayer’s hematoxylin, dehydrated and mounted with a glass coverslip. Positive controls (a tissue known to contain the antigen under study) were included in all reactions in accordance with manufacturer´s protocols. The negative control consisted in omitting the primary antibody and incubating slides with PBS and replacing the primary antibody with normal serum. Quantification for e-cadherin and vimentin were done using ImageJ to measure relative DAB/nuclear signal in 10 randomly selected fields.

### Statistical analysis

All data was presented as mean ± SEM using the software GraphPad Prism 7.0 (GraphPad Software Inc., San Diego, USA). The alpha error was established at 5% and 95% confidence interval. Statistical differences on cell proliferation, migration and colony formation were determined by the test two-way Anova with Tukey's multiple comparisons test. The significance in cell migration was analysed by multiple Student’s t test for paired samples. Data were deemed to be statistically significant if *P* < 0.05.

## Results

### E-liquid exposure changes oral epithelial cell morphology, survival and increases cell death

**Figure 2:**
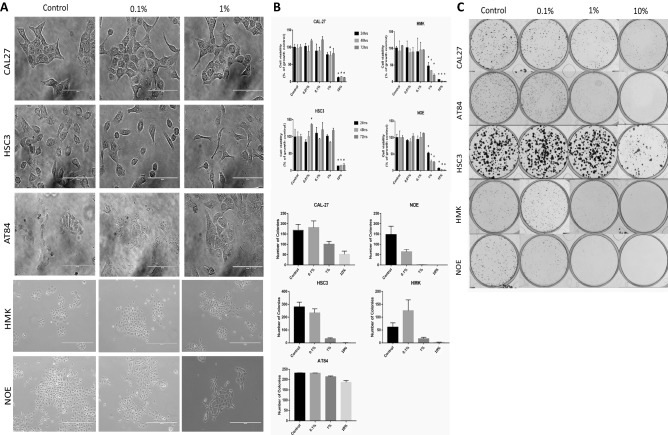
E-liquid exposure promotes change in cell morphology and survival of normal and malignant oral epithelial cells. (**A**) Representative images of the OSCC cell lines (CAL27, HSC3 and AT84) and normal oral epithelium cells (HMK and NOE) cell cultures treated at 0.1% (0.2 mg/mL) of e-liquid for 72 h before trypan blue staining. Magnification 200X illustrating changes in cell morphology. (**B**) E-liquid exposure resulted in increased cell death under concentrations above 1% for normal cells and 10% for OSCC cells. Results are shown as the mean percentage of cell death per sample ± SEM. Magnification 200X. (**C**) Colony counts were normalized to the untreated control cell cultures. Graphed results are given as mean colony count ± SEM. *represents significant difference between the control group*P < 0.0001.

The OSCC cell lines (CAL27, HSC3 and AT84) and normal oral epithelium cells (HMK and NOE) were treated with 0.01% to 10% (v/v) e-liquid concentration for 24 h, 48 h and 72 h and evaluated for cell morphology and viability evaluation (Fig. 2A,B). E-liquid exposure induced morphological cell changes, leading to cell detachment and loss of confluency. Representative images of OSCC cell lines treated at non-lethal concentration of 0.1% (v/v) of e-liquid showed significant alteration in cell morphology compared to the control (Fig. 2A). However, there was an overall decrease in cell viability when samples were treated with high concentrations of e-liquid solutions (10% v/v) for a long period (72 h) compared to the untreated control (Fig. 2B). This pattern was differently observed when comparing normal to OSCC cell lines stimulated with > 1% concentration of e-liquid solution. At this high concentration, HMK and NOE presented a significant decrease in cell viability, whereas OSCC cell lines maintained their viability to similar percentages observed in untreated controls (Fig. 2B). At lower concentrations, this differential susceptibility to e-liquid compounds was even more noticed as OSCC cell lines, which demonstrated a slight increase in proliferation as compared to control cells.

To evaluate the survival of morphologically normal oral epithelial and OSCC cells exposed to varying doses of e-liquid treatment, clonogenicity assay was performed using cells treated with 0.1 and 1% e-liquid solutions (Fig. 2C). Tumor cells (CAL27, HSC3 and AT84) and normal cell lines (HMK and NOE) were treated for 7 days prior to colony enumeration. OSCC cell lines demonstrated a more resistant phenotype to e-liquid-induced cell death when compared to normal cells. In NOE, 1% by volume of e-liquid treatment resulted in 100% cell death. Our results also revealed a stepwise decrease in colony count and survival with increasing e-liquid doses (in both flavored and unflavored e-liquid), with a greater than twofold decrease in survival in all cell lines following exposure to only 1% by volume. The total number of colonies detected in OSCC cell lines were merely affected by the e-liquid treatment (Fig. 2).

### E-liquid increases cell migration and tumor invasion

**Figure 3:**
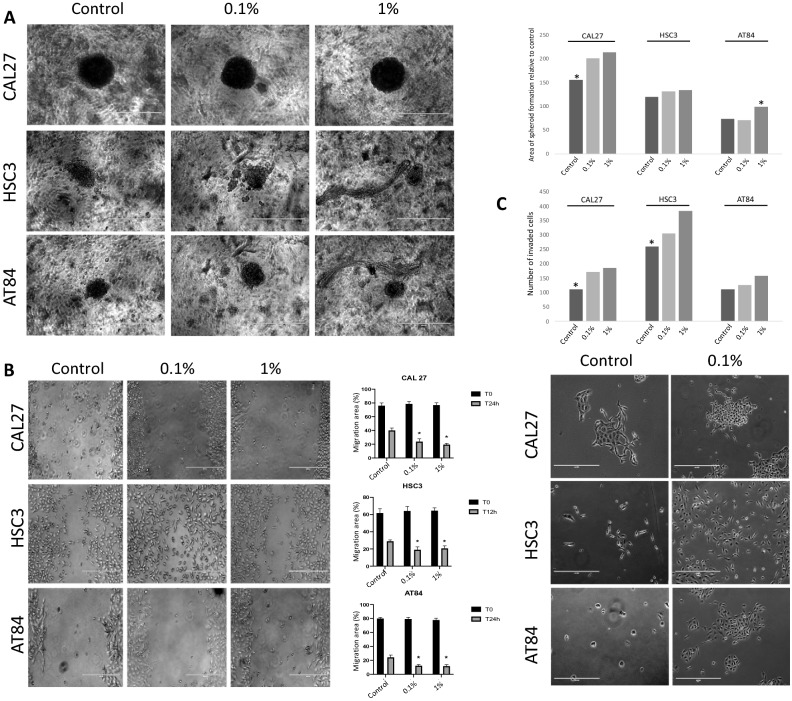
E-liquid increases anchorage-independent cell growth and promotes tumor migration and invasion in OSCC cells. (**A**) E-liquid treatment increased the number and size of spheroid formation in semisolid media. CAL27, HSC3 and AT84 cell lines were able to survive after E-liquid treatment and exhibited anchorage-independent growth (**B**) Wound healing assays show that e-liquid stimulation with 0.1% or 1% nicotine can promote migration and invasion of OSCC quiescent cells. Cells treated with media containing 10% FBS were used as the positive control for the assay. E-liquid induced the migration and invasion of the cells in a dose-dependent manner. (**C**) E-liquid was able to potently promote invasion of OSCC cells at a concentration of 0.1% and 1% as seen in a Boyden-chamber assay. E-liquid induced the migration and invasion of the cells in a dose-dependent manner. *P < 0.0001.

Since e-liquid treatment induces an increase proliferation of OSCC cell lines (CAL27, HSC3 and AT84) and significantly modify the morphology of normal oral epithelium cells (HMK and NOE), we investigated the impact of e-liquid on oral cancer cell adherence-independent growth (Fig. 3A). The ability of cells to grow independently of adhesion is a feature of aggressive cancer cells. As shown in Fig. 3A, the treatment with e-liquid favored the colony-forming capacity in semisolid media. OSCC cells were able to survive and increased the number as well as the size of the colonies in the absence of anchorage after E-liquid treatment. Next, critical steps in the progression of cancer to metastatic disease were assessed to evaluate the impact of e-liquid on motile and invasive properties of OSCC cells. Cells were treated with 0.1%, 1% nicotine for 24 h; treatment with 10% serum was used as a positive control. E-liquid treatment enhanced the migration and invasion capability of CAL27, HSC3 and AT84 cells in a dose-dependent manner; the maximum effect was observed at 1% (*P < 0.001) (Fig. 3C).

### E-liquid induces EMT and increases metastatic competence in OSCC

**Figure 4:**
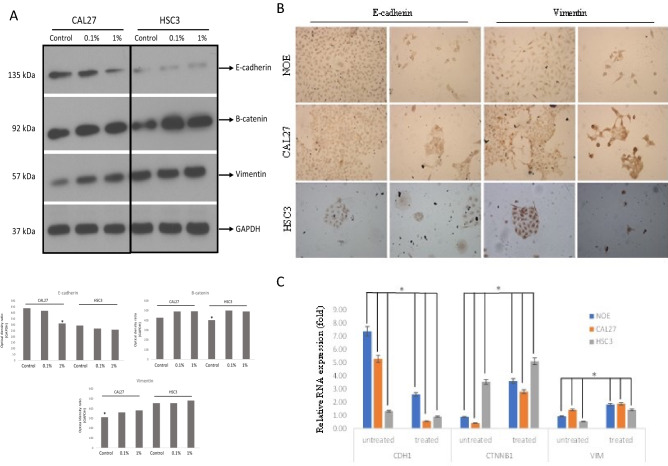
E-liquid induced epithelial to mesenchymal transition (EMT) in normal epithelium oral cells and OSCC. (**A**) Treatment with 0.1% and 1% of e-liquid induced down-regulation of epithelial markers E-cadherin, whereas it caused concomitant increase of mesenchymal proteins and B-catenin and vimentin in CAL27 and HSC3 human OSCC cell lines. GAPDH was used as the control for the assay. Original gels/blots are used in figures are available in the supplementary material provided to the journal. (**B**) Immunocytochemistry reaction showing changes in morphology before and after treatment with 1% e-liquid in normal epithelial cells (NOE) and OSCC cell lines (CAL27 and HSC3). (**C**) Quantitative RT-PCR (q-RT-PCR) showing that gene expression for *CDH1*, *CTNNB1* and *VIM* had a significant difference after e-liquid treatment. *GAPDH*, *HPRT*, and *ACTB* were used as reference genes. *P < 0.0001.

Since e-liquid can promote the invasive properties of OSCC, we evaluated the signaling pathways underlying the pro-invasive activity of e-liquid. Once the cell morphology was clearly changed after e-liquid treatment, quantification for E-cadherin, B-catenin, and vimentin proteins as well as their respective genes were then performed to specifically assess EMT processes. These proteins are involved in tumor cell plasticity, attachment, migration, and signaling. Immunocytochemistry and immunoblotting assays demonstrated that e-liquid treatment induced EMT process by modulating the expression of the proteins (Fig. 4A,B). OSCC cell lines (CAL27, HSC3) and normal cells (NOE) were treated with 0.1 and 1% e-liquid solutions for 48 h. Each of these cell lines has different behaviour in preclinical and animal models. CAL27, a well-differentiated OSCC cell line showed significant change in the morphology (more fibroblast-like) after being exposed to e-liquid (Fig. 2A). Compared to the metastatic HSC3 cells, CAL27 cells showed a similar invasive behaviour after the e-liquid treatment, with a significant change in gene and proteins implicated in cancer cell aggressiveness (Figs. 3, 4). Even at the low doses of e-liquid treatment, a significant down-expression of E-cadherin was seen in OSCC cells (CAL27 and HSC3) and also NOE normal cells (Fig. 4A,B). In addition, in HSC3 cells exposed to 0.1% e-liquid showed an upregulation of B-catenin, but not vimentin protein levels (Fig. 4A). Quantitative RT-PCR (q-RT-PCR) analysis further revealed the down-regulation of e-cadherin (*CDH1*), and overexpression of B-catenin (*CTNNB1*) and vimentin (*VIM*) gene expression in cells exposed to 0.1% e-liquid (Fig. 4C). The enhanced invasive migratory activity associated with the loss of epithelial markers, such as E-cadherin, and the acquisition of mesenchymal markers such as vimentin and B-catenin, strongly suggest that epithelial cells are undergoing to an aggressive phenotype within the framework of epithelial-mesenchymal transition (EMT).

## Discussion

While cigarette smoking is recognized as the major risk factor for oral cancer, recent studies suggested that e-cigarette may also induce the signaling pathways associated with tumorigenesis, therefore impacting oral healthy cell microenvironment^17,18,22,23^. Our study was intended as a response to the priority expressed by the clinicians on potential hazard risk of e-cigarettes in particular by promoting oral cancer development, progression and metastasis.

The direct health impact of exposure to conventional cigarettes in oral tumor progression is well established with approximately 75% of OSCC being attributable to tobacco smoking and alcohol^24^. The relationship with tobacco and the development of head and neck squamous cell carcinoma is explained by the fact that conventional cigarettes result in high exposure to nitrosamines and polycyclic hydrocarbons, which are the main genotoxic agents^25^. The presence of these substances in tobacco causes multiple mutations, including in the *TP53* tumor suppressor gene. These mutations occur more frequently in smokers compared to non-smokers diagnosed with the same tumor^26^. The use of conventional cigarettes increases tumor aggressiveness by stimulating tumor cell proliferation, angiogenesis and migration, as well as reducing the patient's response to radiotherapy and chemotherapy^27,28^. Several prevention programs around the world have proposed e-cigarettes as an alternative to safety replacing conventional cigarettes. However, e‐cigarette research is still lagging considering the popularity with vapers and long-term safety of these new products. E-devices generate substantial amounts of fine particulate matter, toxins and heavy metals at levels that can exceed those observed for conventional cigarettes^29^. Furthermore, there is scientific evidence to support that e-cigarette may pose the most seriously harm to health, e.g. increasing the risk of cancer and heart diseases^30^. Indeed, this study shows molecular alterations linked with the effects of chemical mixture generated by e-cigarettes and their impact at oral cancer progression and metastatic competence.

Head and neck cancers are remarkably heterogeneous comprising several subtypes with distinct pathological and molecular features and can occur in different anatomic sites, including oral cavity (lips, buccal mucosa, hard palate, anterior tongue, floor of mouth and retromolar trigone), nasopharynx, oropharynx (palantine tonsils, lingual tonsils, base of tongue, soft palate, uvula and posterior pharyngeal wall), hypopharynx (the bottom part of the throat, extending from the hyoid bone to the cricoid cartilage) and larynx. of the upper aerodigestive tracts. Oral cavity, hypopharynx and larynx cancers are primarily associated with tobacco smoke, alcohol abuse or both, whereas palantine and lingual tonsils of the pharynx cancers are increasingly attributed to infection with human papillomavirus (HPV), primarily HPV-16. Both the carcinogenic process and progression of initiated cancer depend on anatomical location, histologic configuration (whose thickness and degree of keratinization depend on the location and tissue microenvironment requirements), and aetiological agent (tobacco-specific chemical carcinogens versus virus). Currently, there is no study correlating the use of e-cigarette and incidence rates based on the anatomical cancer site and/or gender differences.

Using a panel of normal and tumor cell lines (metastatic and non-metastatic OSCC), we demonstrated that e-cigarettes can mediate tumor progression and metastasis by inducing EMT phenotype, which enhances the invasive abilities of OSCC cells and has potential to promote changes in cell morphology and behaviour in normal oral keratinocytes. In the EMT, epithelial cells reorganize their cytoskeleton properties (down-regulation of epithelial markers e.g., E-cadherin] and up-regulation of mesenchymal markers [e.g., vimentin]), undergo a change in the signaling network that defines a mesenchymal phenotype (e.g., TGFβ, Wnt) and reprogram gene expression (e.g., the transcriptional factors: Snail, Twist1). These changes increase cell motility and enable the development of an invasive phenotype^31–37^. Traditional cigarette smoking increases inflammation and oxidative stress which accelerate cellular senescence and induce cell proliferation^38–40^. Numerous compounds in the e-cigarette form reactive oxygen species (ROS) capable to promote DNA mutations and genetic instability^16–21^. These events potentially contributing to various aspects of cancer cell behavior, including cell proliferation, survival, invasion, angiogenesis and metastasis, which are also associated with the acquisition of EMT phenotype. Previous in vitro studies indicated that treatment with nicotine promotes EMT and increases cytokine production potentializing inflammation and lung cancer progression^38^. Furthermore, patients with low grade small-cell lung cancer who continue to smoke during chemotherapy and radiotherapy have poorer survival compared with those who do not^39^. Recent studies have shown that toxic and carcinogenic substances are present in the e-liquid vapor can reach levels equal to (e.g., acetaldehyde and chromium) or higher (e.g., formaldehyde and nickel) compared to conventional cigarette smoke to the detriment of increased device power, polycyclic aromatic carbides, tobacco alkaloids, heavy metals and flavors^15,40,41^. In this way, E-cigarettes can not be considered as a smoking cessation product. Among all of the alternative tobacco products, e-cigarettes are the least regulated^42^. Clinicians should act on the side of caution when advising patients about the use of e-cigarettes. The ongoing popularity of e-cigarettes and the continued evolution in e-cigarette like devices require a careful evaluation of the present evidence and honest discussions with patients about potential risks to consider.

Moreover, given the immense variability of e-cigarettes designs and usage habits, these potential health risks may even be simplistic. Regulation, quality control standards, and safety assessments for e-cigarettes products have become further complicated by the rapid evolution of new devices and e-liquid formulations. The newer generations of e-cigarettes often boast larger batteries with adjustable voltages and a wider range of flavours, thus appealing more broadly to vapers^43,44^. Next-generation models have been shown to deliver greater doses of nicotine to users, as well as higher levels of hazardous carbonyls and other toxins with increasing voltages^44–46^. Varied e-cigarettes usage patterns, such as different puff durations or average “vaping” session lengths, have also been associated with differential absorption of nicotine^47^. These trends in e-cigarettes use and design further diversify hazard risks that must be undertaken to accurately capture all health risks experienced by vapers. While focused safety assessments still remain elusive for most of the e-cigarette’s user demographic, our study nevertheless suggests that e-cigarettes should be viewed as far from risk-free or harmless in the interim.

## Conclusion

Lack of long-term prospective and large-scale case–control studies is a major limiting factor in assessing the association of e-cigarettes with oral cancer progression. In addition, comparative analysis suggesting a relatively lower toxic level of e-cigarette compared to combustible cigarettes, creates a misconceived notion on the safety of e-cigarette use. It is important to acknowledge that a relatively lower toxic dosage does not mean it is risk-free. Evidence of the presence of carcinogenic agents in e-liquid and their vapor inducing DNA strand breaks and gene alteration is compelling evidence, but not sufficient to infer a causal relationship.

Clearly experimental validation studies involving animal model and/or clinical sampling of oral biopsy tissues are needed to assess if the relatively lower dosage of specific carcinogens released by the e-cigarette (e.g. tobacco-specific nitrosamines) can induce malignant transformation in oral epithelial cells or promote tumor progression by inducing EMT. Delineating the molecular pathway of e-cigarette-induced oral mucosal changes could provide further valuable insight into e-liquid’s oral carcinogenesis and aid in identifying mechanistic insights into the action of nitrosamine carcinogens, and other important volatile reactive chemicals present in the e-cigarette aerosol. In addition, longitudinal clinical studies with long-term follow-up are needed to discriminate e-cigarette *versus* other associated risk and genetic predisposition factors that may contribute to oral carcinogenesis.

## Supplementary Information

Supplementary Figures.

## Data Availability

The datasets used and/or analysed during the current study are available from the corresponding author on reasonable request.
