# Supplementary material for: E-liquid alters oral epithelial cell function to promote epithelial to mesenchymal transition and invasiveness in preclinical oral squamous cell carcinoma
Source: Sci Rep. 2023 Feb 27;13:3330. doi: 10.1038/s41598-023-30016-0 (PMC9971414; doi:10.1038/s41598-023-30016-0)

Western Blot – Figure 4 – Panel A– E-liquid alters oral epithelial cell function to promote epithelial to mesenchymal transition and invasiveness in preclinical oral squamous cell carcinoma

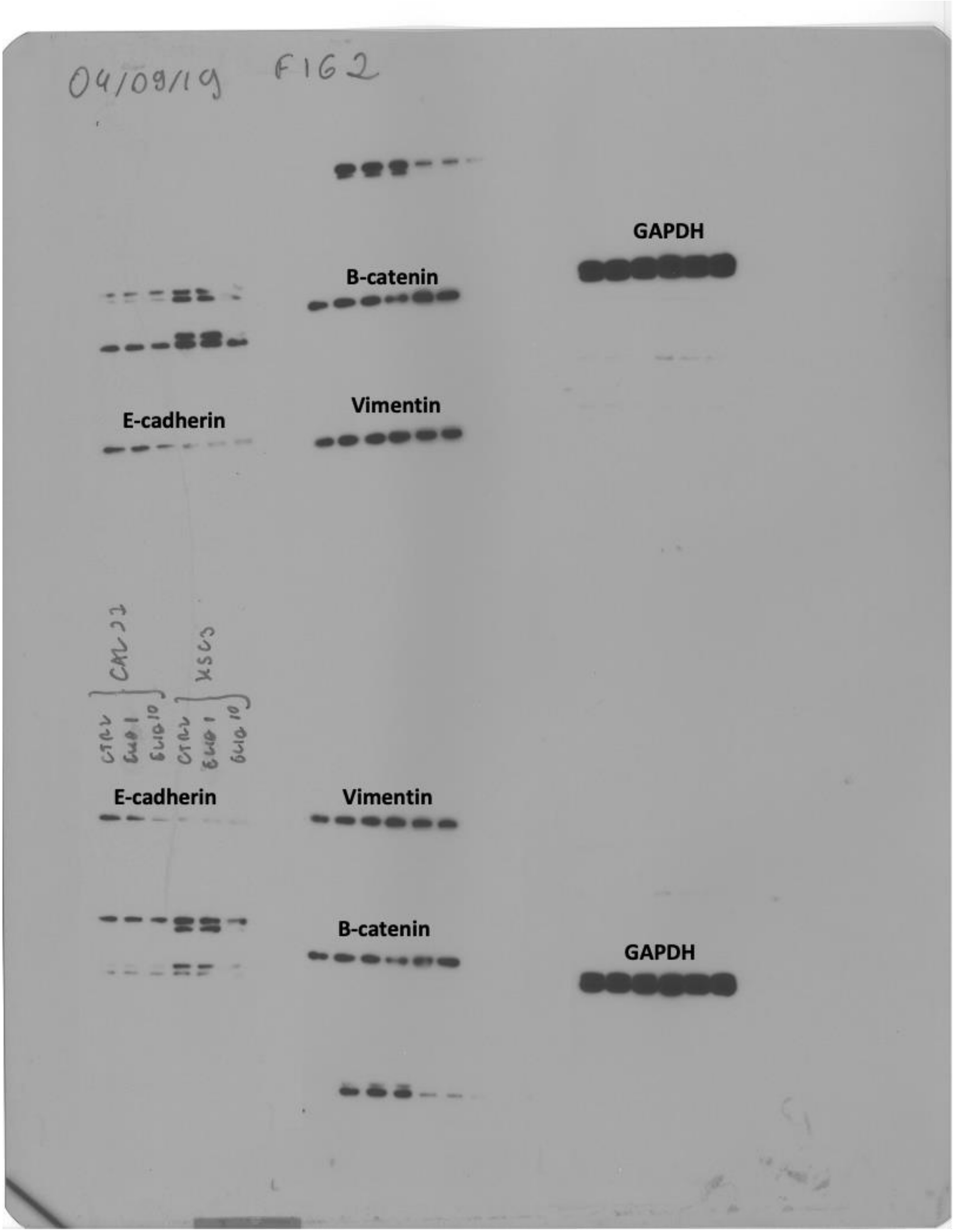

Supplement: Supplementary file 1 — Supplementary Figures. [file 41598_2023_30016_MOESM1_ESM.pdf]
